# A Cooperative Coevolutionary Approach to Discretization-Based Feature Selection for High-Dimensional Data

**DOI:** 10.3390/e22060613

**Published:** 2020-06-01

**Authors:** Yu Zhou, Junhao Kang, Xiao Zhang

**Affiliations:** 1 College of Computer Science and Software Engineering, Shenzhen University, Shenzhen 518060, China; yu.zhou@szu.edu.cn (Y.Z.); 1800271054@email.szu.edu.cn (J.K.); 2 College of Computer Science, South-Central University for Nationalities, Wuhan 430074, China; 3 Hubei Provincial Engineering Research Center for Intelligent Management of Manufacturing Enterprises, Wuhan 430074, China

**Keywords:** feature selection, genetic algorithms, particle swarm optimization, cooperative coevolutionary, entropy-based cut-points

## Abstract

Recent discretization-based feature selection methods show great advantages by introducing the entropy-based cut-points for features to integrate discretization and feature selection into one stage for high-dimensional data. However, current methods usually consider the individual features independently, ignoring the interaction between features with cut-points and those without cut-points, which results in information loss. In this paper, we propose a cooperative coevolutionary algorithm based on the genetic algorithm (GA) and particle swarm optimization (PSO), which searches for the feature subsets with and without entropy-based cut-points simultaneously. For the features with cut-points, a ranking mechanism is used to control the probability of mutation and crossover in GA. In addition, a binary-coded PSO is applied to update the indices of the selected features without cut-points. Experimental results on 10 real datasets verify the effectiveness of our algorithm in classification accuracy compared with several state-of-the-art competitors.

## 1. Introduction

Feature selection (FS) is an important task in machine learning, aiming to find an optimal subset of features to improve the performances of classification [1] or clustering [2,3]. By removing those redundant and irrelevant features, the model complexity is reduced and the overfitting in the training process can be avoided. Current FS algorithms can be generally categorized into wrapper and filter methods [4]. The filter approaches select the feature subset by scoring the features, and set a threshold to select those features that meet the conditions to form the feature subset. The wrapper methods treat FS as a search problem, generating different feature subsets and then evaluating the feature subsets until a certain feature subset reaches the expected standard. In general, the wrapper methods can achieve better and more robust results than the filter methods, but suffering from heavy computational burden.

For wrapper feature selection, it is essentially a combinatorial optimization problem, where the search space for data with *N* features is $2^{N}$. For high-dimensional data, the search space rises exponentially [5]. In the past few decades, it has been empirically demonstrated that evolutionary algorithms achieved their success in many different applications, for example soft sensors [6] and compressed sensing [7]. To overcome the shortage of traditional search methods [8,9], evolutionary algorithms were introduced into FS, among which, particle swarm optimization (PSO) [10,11,12] and genetic algorithms (GA) [13,14] are most widely adopted, due to their fast convergence [15] and powerful search capabilities [16], respectively. The core task of FS performed by evolutionary algorithms (EAs) is to identify the indices of selected features, so discretization-based encoding in evolutionary algorithm is desirable. The most commonly-used strategy is to apply a binary coding that indicates whether a feature is selected or not (one or zero), such as binary PSO (BPSO) [17]. However, for high-dimensional data, the search process is easily trapped into local optima. Another approach is to encode the indices of selected features directly. However, this often suffers from different encoding lengths and complicated updating rules during the search procedure, which is not very appropriate for the high-dimensional FS problem.

Recently, discretization-based feature selection algorithms have received much attention due to their good performance in high-dimensional data classification [18,19,20,21]. These algorithms map the search features into that of cut-points in PSO, which are generated by the univariate discretization algorithm MDL [22], combining feature discretization and feature selection into one stage. However, current discretization-based FS methods treat each feature independently without taking into account feature interaction, where the features without cut-points are not involved in the search process, leading to information loss and limiting the classification accuracy.

In recent years, the idea of coevolution has been successfully applied in different applications involving a large number of design variables, such as classification [23], artificial neural networks [24], function optimization [25], and image processing [26]. Cooperative coevolutionary algorithms (CCAs) decompose the original problem into subproblems with less decision variables. During the optimization process, CCAs are usually composed of two or more populations, which evolve simultaneously by applying different objectives or search methods and allow interaction, trying to obtain a global solution after combining the respective final solutions together. More related works along CCA can be found in [27].

Inspired by this methodology, in this paper, we propose a cooperative coevolutionary discretization-based FS algorithm (CC-DFS), which searches the subset of features with cut-points and without cut-points simultaneously. In our method, at first, the discretization technique is used to obtain the features with and without cut-points. Then, GA is applied to search the features with cut-points where a reset operation is used to jump out of local optima, and an individual scoring mechanism is introduced to control the probability of crossover and mutation. For features without cut-points, a binary-coded PSO is applied to update the indices of the selected features without cut-points. The final selected feature subset is composed of the results obtained by evolving both populations. Experimental studies on 10 real-world datasets demonstrate the superiority of our proposed approach in classification accuracy compared with some state-of-the-art discretization-based FS methods.

## 2. Background

### 2.1. Particle Swarm Optimization

PSO [28] is a population-based stochastic algorithm that searches the decision space by simulating the flight of birds in nature. In PSO, the population maintains a set of particles, each of which represents a feasible solution in the decision space. A fitness function of these particles is used to guide the search. The particle with the best fitness value in the population is selected as gbest, and the best individual fitness value achieved by each particle will be recorded as pbest. Each particle has a position vector and a velocity parameter, which are randomly initialized. The velocity parameter is updated according to pbest and gbest in each iteration as follows:

(1) $$v_{id}^{t+1}=w\times v_{id}^{t}+c_{1}\times rand\times \left(p_{id}^{t}-x_{id}^{t}\right)+c_{2}\times rand\times \left(g^{t}-x_{id}^{t}\right)$$

(2) $$\begin{array}{c} x_{id}^{t+1}=x_{id}^{t}+v_{id}^{t+1} \end{array}$$

where $x_{id}^{t}$ and $v_{id}^{t}$ represent the position and velocity of particle *i* in dimension *d* at the $t^{\mathrm{th}}$ iteration, *w* represents the inertia weight, which indicates the effect of the current velocity on the updated velocity, c1and c2 are acceleration coefficients used to control the effects of pbest and gbest, and rand is a function used to generate random numbers between zero and one. The velocity is usually limited by a preset threshold $V_{max}$, which limits the velocity to [$-V_{max}$, $V_{max}$].

### 2.2. Genetic Algorithm

**Figure 1:**
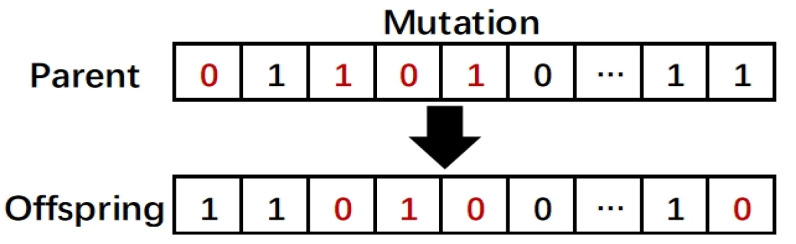
Bit-flip mutation. Each gene of an individual has a certain probability to perform the flip operation.

**Figure 2:**
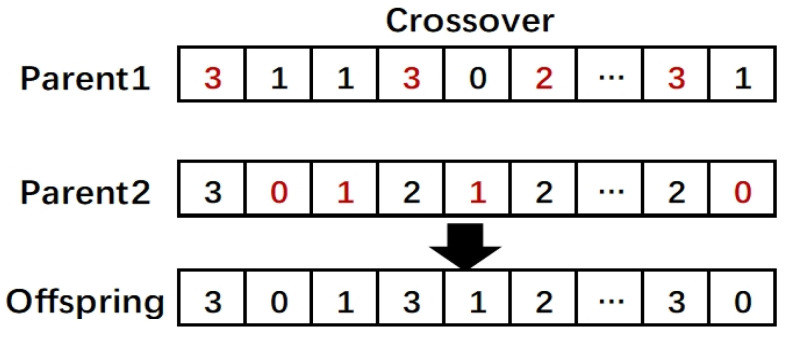
Discrete crossover. Two individuals X and Y are selected as parents, and genes are selected from the parents to produce offspring.

The genetic algorithm (GA) [29] is a heuristic search algorithm inspired by natural selection and biological inheritance, which provides a powerful search capability to find near optimal solutions in complex and large search spaces [16]. GA maintains a set of candidate solutions in a population, which are used to explore the decision space. Individuals (or chromosomes) in a population represent a set of feasible solutions to the problem, and next-generation individuals are produced through operators such as crossover and mutation. In addition, a fitness function is used to evaluate the quality of the individual, and those individuals with better fitness function values are selected to participate in the next iteration process. The bit-flip mutation [30] and discrete crossover [31] operations are shown in Figure 1 and Figure 2, respectively. In bit-flip mutation, a parent with binary encoding randomly selects a gene to flip it from zero to one, and vice versa. In discrete crossover, two parents are randomly selected to generate one offspring, and the offspring randomly selects genes from both parents.

### 2.3. Minimum Description Length Principle

The minimum description length (MDL) [22] is a supervised multivariate discrete algorithm, which finds the cut-points that satisfy the minimum description length principle (MDLP) to discretize the data. For each feature *A*, the features are sorted according to the feature value, and the algorithm selects the candidate cut-points, the feature value of which lies between instances of different classes. The formula for calculating the information gain of the cut-point *T* is as follows:

(3) $$\mathrm{Gain}(T,A;S)=E(S)-\frac{|S_{1}|}{\left|S\right|}E\left(S_{1}\right)-\frac{|S_{2}|}{\left|S\right|}E\left(S_{2}\right)$$

where E(S) denotes the entropy of dataset *S* and $\left|S_{1}\right|$ and $\left|S_{2}\right|$ represent the number of samples of each part after the dataset *S* is divided into two sample subsets by the cut-point *T*.

The algorithm divides the dataset recursively until the cut-point cannot pass the MDLP criterion. Feature values that satisfy the MDLP criteria are used as our cut-points to discrete datasets. MDLP criteria are calculated based on Equation (4).

(4) $$Gain\left(T,A;S\right)>\frac{\log_{2}\left(|S|-1\right)}{\left|S\right|}+\frac{\delta (T,A;S)}{\left|S\right|}$$

δ(T,A;S) is calculated by (5).

(5) $$\delta \left(T\;,A;S\right)\;=\;\log_{2}\left(3^{k_{s}}\;-\;2\right)\;-\;\left[k_{s}E(S)-k_{s_{1}}\;E\left(S_{1}\right)\;-\;k_{s_{2}}E\left(S_{2}\right)\right]$$

where $K_{s}$ denotes the number of classes present in *S* and $S_{1}$ and $S_{2}$ represent the two sample subsets after the sample *S* is divided.

### 2.4. Discretization-Based FS Algorithms

In the past few years, discretization-based algorithms have demonstrated great potential to deal with high-dimensional data, such as EPSO [19] and PPSO [18].

**Figure 3:**
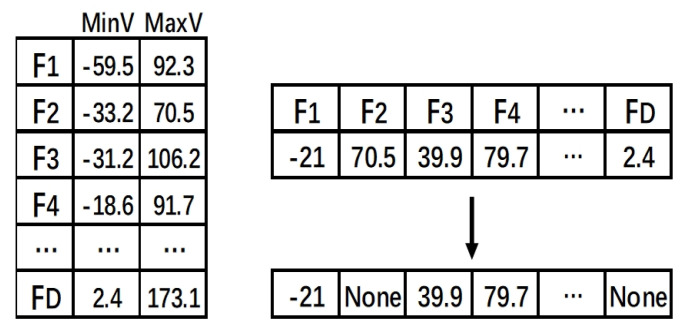
The particle representation of EPSO.

In EPSO, individuals in the population are encoded as real numbers that are within the range of feature values. As shown in Figure 3, the particle’s position of $F_{1}$ is −21, falling within its feature value range, which represents selecting feature F1 and using this value to discretize the corresponding feature.

During the update process, if the value of the particle in a dimension exceeds the upper or lower limit of the feature, the value is set to the corresponding upper or lower limit. When the value of the particle in a dimension is equal to the upper or lower limit, the feature is discarded, because when the feature is discretized with the upper limit or the lower limit of the feature value, the discrete values of the feature are equal, which means that it cannot contribute to classification.

**Figure 4:**
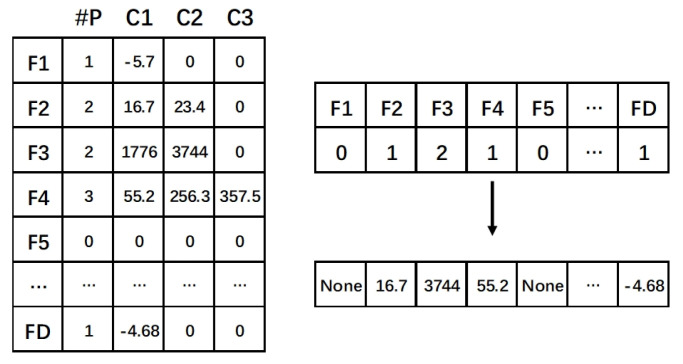
The particle representation of PPSO.

EPSO directly uses features to evolve the cut-points, which results in a huge search space. PPSO introduced the MDL algorithm to select the features with cut-points and encode the position of cut-points in the search space of PSO. Individuals in the population are encoded by the cut-point table, as shown in Figure 4. The MDL algorithm was initially used to calculate the cut-point table of features as shown on the left side, where #P represents the number of cut-points for each feature, and $C_{1}$, $C_{2}$, and $C_{3}$ represent the index of the cut-point for the features. If the value exceeds the range of the cut-point index during the particle update process, it is set to zero, which means the feature is not selected. For instance, the value of F3 is two, which indicates that the cut-point C2 for F3 is selected to discretize the feature; the value for F1 is zero, which means that the feature is discarded.

## 3. Proposed Method

**Figure 5:**
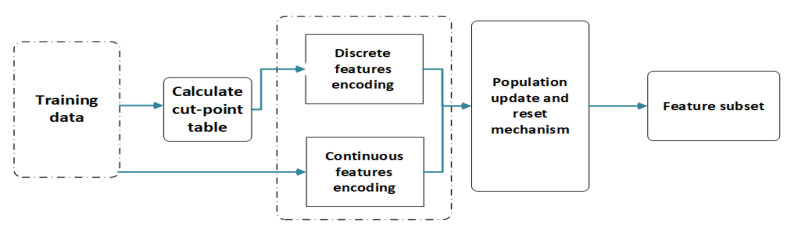
Overview of our proposed method.

Although discretization-based FS algorithms have achieved good results in high-dimensional data, the absence of features without cut-points causes information loss, which affects the classification accuracy negatively. The discretization process treats each feature independently, and some groups or pairs of weak features may have a greater impact on the classifying performances than one individual strong feature; therefore, features that do not have cut-points or participate in the discrete process are likely to contribute to the classification task. In this section, we introduce the details of our proposed cooperative coevolutionary discretization-based FS (CC-DFS) method, which considers searching the feature subset from the features with and without cut-points simultaneously. The pseudocode of CC-DFS is presented in Algorithm 1, and Figure 5 shows a flowchart of our algorithm.
**Algorithm 1:** The pseudocode of the proposed CC-DFS.
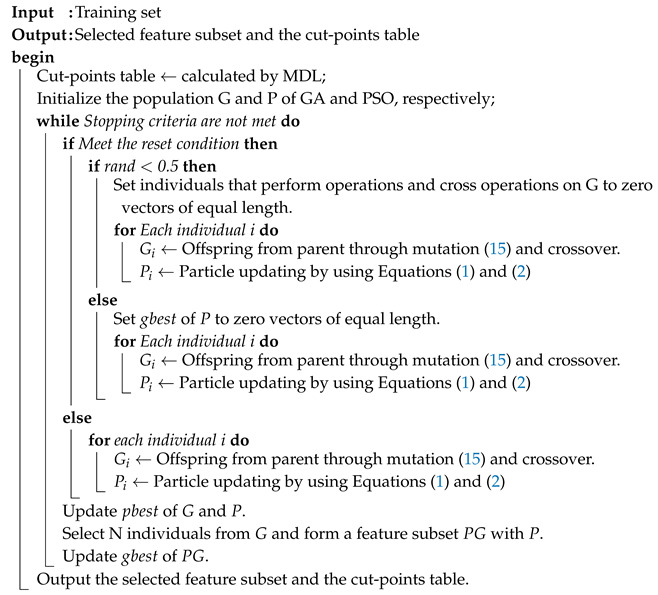


### 3.1. Representation of an Individual

**Figure 6:**
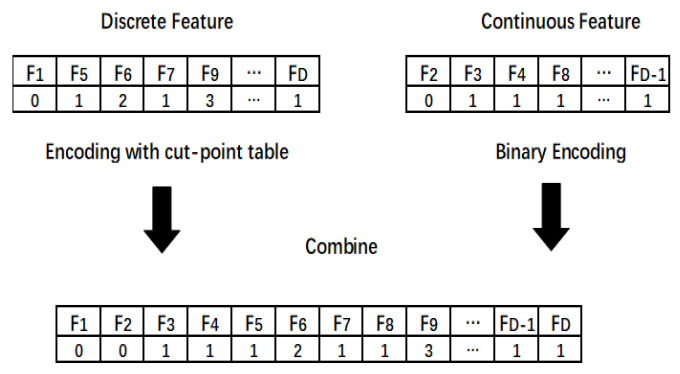
The particle’s representation of our algorithm.

CC-DFS was designed to search both the features with and without cut-points, simultaneously. For simplicity, we denote the features with cut-points by discrete features and the features without cut-points by continuous features. To achieve this, the whole decision space A is divided into two parts, where decision space D contains discrete features, while decision space C contains continuous features. The population is encoded using two different encoding methods where the discrete features use the encoding way of PPSO and the continuous features use binary encoding. As shown in Figure 6, for feature F5, one means to select the cut-point with Index 1 in the cut-point table to discretize the data and for F6; it means selecting the cut-point with Index 2 in the cut-point table. The features with a value of zero mean not selecting a cut-point to discretize the feature and for continuous features, and zero means discarding the feature, while one means selecting the feature. At last, these two parts are combined into one vector to represent a feature subset.

### 3.2. Fitness Function

The fitness function plays a vital role in the population update process. It is used to evaluate and guide the population update. In the FS problem, we want to use fewer features to maintain a better or competitive classification accuracy than all the features. Considering the above issues, we use the distance [32] and $balanced_{-}error$ to guide individual updates. These two objective functions use weight aggregation [33,34,35] as the fitness function as shown in (6), where the smaller the fitness value, the better the individual’s performance.

(6) $$fitness=(\beta \times balanced_{-}error+\left(1-\beta \right)\times distance)$$

where β is a weight coefficient to combine the $balanced_{-}error$ and distance. $balanced_{-}error$ and distance are calculated by (7) and (8), respectively.

(7) $$balanced_{-}error=\frac{1}{n}\sum_{i=1}^{n}\frac{FP_{i}}{\left|S_{i}\right|}$$

(8) $$distance=\frac{1}{1+\exp^{-5\left(D_{W}-D_{B}\right)}}$$

(9) $$\begin{array}{c} D_{B}\;=\;\frac{1}{\left|N\right|}\sum_{i=1}^{\left|N\right|}\min_{\left\{j|j\neq i,class\left(V_{i}\right)\neq class\left(V_{j}\right)\right\}}Dis\left(V_{i},V_{j}\right) \end{array}$$

(10) $$D_{W}\;=\;\frac{1}{\left|N\right|}\;\sum_{i=1}^{\left|N\right|}\max_{\left\{j|j\neq i,class\left(V_{i}\right)=class\left(V_{j}\right)\right\}}Dis\left(V_{i},V_{j}\right)$$

where n denotes the number of the classes, FP${}_{i}$ represents the number of misclassified samples in class i, $\left|S_{i}\right|$ means the number of samples in each class, $\left|N\right|$ denotes the number of samples, $D_{B}$ represents the distance between each sample in the data and its nearest sample of different classes, and $D_{W}$ represents the distance between each sample in the data and its farthest sample of the same class.

In CC-DFS, since two different encoding methods for FS are performed for two different decision spaces, the calculation of $Dis(V_{i},V_{j})$ is also different from each other. As $Dis(V_{i},V_{j})$ represents the distance between two feature subset vectors $V_{i}$ and $V_{j}$, for discrete features, the Hamming distance is applied for discrete features, while the Euclidean distance is calculated for continuous features. In addition, the historically optimal positions of discrete and continuous feature subset individuals are recorded as pbest. The population optimal record of the feature subset of discrete and continuous feature combinations is gbest, where its discrete feature part is called dbest as the pbest of discrete individuals and the continuous part cbest as the pbest of continuous feature individuals.

### 3.3. Updating Strategy

For discrete features and continuous features, due to the easy implementation and fast convergence, GA and PSO are employed to search their feature subset, respectively.

#### 3.3.1. Search in Discrete Features

Different from the traditional GA algorithm, we propose a novel mutation and crossover operation. In our method, each individual in the population is scored using a ranking function, where individual scores are used to control their probability of mutation and crossover. Inspired by the ranking function in [36], considering that the population’s variability becomes lower with the increase of iterations, an adaptive scoring mechanism is introduced, which aims to reduce the mutation and crossover probability of each dimension in the later stage shown as follows:

(11) $${\mathrm{Pc}}_{i}=Lmin+Lmax\times \frac{\exp^{\frac{10(r-1)}{S-1}}-1}{\exp^{10}-1}$$

(12) $$Lmax=Lmax-\frac{t}{It}\times Lmax$$

where Lmin and Lmax control the upper and lower limits of the score, *S* represents the number of populations, *r* is the individual’s ranking, *t* is the number of current iterations, and It is the maximum number of iterations. In this paper, the lower the fitness value is, the higher the individual ranks. Each dimension of individuals in the population takes its ranking as a probability to decide whether to perform mutation and crossover operations. It is guaranteed that individuals with high rankings have a lower probability of performing mutation and crossover in each dimension, and individuals with low rankings perform a higher probability of mutation and crossover.

The particle encoding range of discrete features is an integer between [0, # C]; therefore, for the mutation operation, traditional bit-flip mutation is not suitable. For each dimension of the individual, we randomly select two other individuals in the population, and the one with the better fitness value performs the mutation operation with the current individual. The mutation operation is similar to the update operation in [37]. The formula is as follows:

(13) $$ch_{\mathrm{id}}^{t+1}=\left\{\begin{array}{c} N(\mu ,\sigma )\\ {\mathrm{pa}}_{\mathrm{id}}^{t} \end{array}\right.\begin{array}{c} \mathrm{rand}<0.5\\ otherwise \end{array}\;$$

where $ch_{\mathrm{id}}^{t+1}$ represents the value of the individual i in dimension d after t iterations, $pa_{\mathrm{id}}^{t}$ represents the value of parent i in dimension d at the $t^{\mathrm{th}}$ iteration, and the mean of the two values performing the mutation operation as μ; the absolute value of the gap as σ, rand is to generate a random number between [0,1], which represents a 50% probability that each dimension of the individual chooses to use the Gaussian function, and there is a 50% probability that the value of the offspring is directly set to the value of the parent at the current position. In the cross operation, we use the ranking function of each individual of the population as the cross probability of the individual. When each gene of the individual performs the cross operation, two other individuals are randomly selected, and then, the highest ranked individual and the current individual perform a crossover operation. In this way, for higher ranking individuals, the crossover probability is lower; while for lower ranking individuals, the crossover probability is higher.

#### 3.3.2. Search in Continuous Features

For continuous feature search, we use binary coding and then use BPSO to search for the continuous feature subset. The difference between BPSO and traditional PSO is that the position update of the particles no longer depends on the position of the previous moment, but the sigmoid function is used to map the velocity to the probability to determine the value of the particle’s current dimension. The formula is as follows:

(14) $$S_{id}^{t}=\frac{1}{1+e^{-v_{id}^{t}}}$$

(15) $$x_{id}^{t}=\left\{\begin{array}{c} 1\\ 0 \end{array}\right.\begin{array}{c} rand<S_{id}^{t}\\ otherwise \end{array}$$

where $x_{id}^{t}$ and $v_{id}^{t}$ represent the velocity of particle *i* in dimension *d* at the $t^{\mathrm{th}}$ iteration. $S_{id}^{t}$ represents the probability of the $i^{\mathrm{th}}$ feature being selected, and rand is used to generate random numbers between zero and one.

#### 3.3.3. Combination of Continuous and Discrete Features

For discrete features, 2N individuals are generated by crossover and mutation of N individuals, and N individuals of continuous features are still N individuals after updating. Therefore, we need to select N individuals in the GA population to form a new feature subset with continuous features. For these discrete features, we use a distance function to measure the quality of these individuals. The first N individuals with better distance measures are selected, and these individuals and continuous feature individuals form a new feature subset.

#### 3.3.4. Reset Operation

When the optimal fitness value of the population has not been improved after three consecutive updates, we consider that the population has fallen into a local optimum. The reset operation is introduced, and in the next update, GA and PSO will randomly choose one to perform the reset operations. If GA is selected, for each individual in the population, the individual who performs mutation and crossover operations with it will be a zero vector of equal length. If PSO is selected, gbest will be set to zero in the next update. Moreover, if the particle’s optimal fitness value has not been improved after 11 updates, the iterative process will stop to reduce the program’s running time.

## 4. Experimental Results and Analysis

This section introduces the details of the experiment and parameters in the algorithm. Besides, the baseline methods for comparison with our method are introduced.

### 4.1. Datasets

Ten real genetic data were used to test the performances of different algorithms, which can be downloaded from https://github.com/primekangkang/Genedata. Table 1 shows the number of features, samples, classes, and the proportion of the smallest class and largest class. It can be observed that most of the data were small samples and high-dimensional, and there were class imbalances. Data were normalized before input.

### 4.2. Parameter Settings and Comparison Method

The parameters of our algorithm are shown in Table 2.

To test the performance of the algorithm, we used the K-NN algorithm with K = 1 as the classifier. The feature subsets selected by the algorithm were input into the classifier to obtain the average classification accuracy on the test set. In the experiment, we conducted a two layer 10-fold cross-validation. At first, the whole dataset was divided into ten portions where one portion was used for testing. Then, the other nine were divided into ten portions again, nine for training and one for validation to calculate the training accuracy. The average testing accuracy was recorded after running the algorithms 30 times.

The classification result of all feature input KNNwas used for comparison with our algorithm. EPSO, deep multilayer perceptron (DMLP), PPSO, and PSO-FS [18] were used to compare with our algorithm, and the parameters of the algorithms used for comparison were the parameters recommended in [18]. For DMLP, the number of neurons in the input layer was set to the number of features, and the number of neurons in the output layer was fixed at 128 as the number of selected features. In addition, the cooperative coevolutionary using bare-bone PSO instead of GA, called CCB-DFS, was also used for comparison, and the parameter setting was the same as CC-DFS.

### 4.3. Results and Analysis

In this section, we compare and analyze the results of our algorithm with respect to other algorithms. The results are shown in Table 3, where Full means we used all feature input KNN for classification and *S* represents the result of the statistical Wilcoxon significance test with a 5% significance level, and the result of the statistical Wilcoxon significance test was a comparison of our method with other methods, where “+” indicates that our algorithm results were better than the compared algorithms, “−” indicates that our algorithm results were worse than the compared algorithms, and “=” indicates that the results of the two algorithms were not significantly different.

#### 4.3.1. CC-DFS VS Full

As can be seen from Table 3, our algorithm selected fewer features, and the classification performance was higher than using all the features on all datasets. For SRBCT, CC-DFS selected 9.5% of all features, with the average classification accuracy of 98.9%, which was an 11.1% improvement over Full, and the best classification accuracy reached 100%. For Leukemia1, three-point-one percent of the features were selected, and the average classification accuracy was 94.02%. Compared with Full, the average classification accuracy was improved by 21.94%, and the best classification accuracy was 97.50%. For 11Tumor, the average classification accuracy increased by 13.42%, and the best classification accuracy increased by 16.35%.

In general, CC-DFS obtained ten “+” compared to Full, which meant that the classification performance was better than Full on all datasets.

#### 4.3.2. CC-DFS vs. DMLP

Compared with DMLP, on eight datasets, our algorithm’s performance was better than DMLP in average classification accuracy. For Prostate, Compared with DMLP, the number of features increased by 52.6, but the average classification accuracy and the best classification accuracy were improved by 14.27 % and 8.77 %, respectively. For Leukemia1, although the best classification accuracy of DMLP was improved by 0.42% compared to CC-DFS, the average classification accuracy was reduced by 2.38%. For Brain2, although the average classification accuracy of DMLP was reduced by 1.71% compared to CC-DFS, the best classification accuracy was improved by 1.94%. Compared with DMLP, CC-DFS obtained eight “+”, one “=”, and one “−”.

#### 4.3.3. CC-DFS vs. PSO-FS

Compared with PSO-FS, for seven datasets, our algorithm selected fewer features, and the classification performance was higher. For the remaining three datasets, although the feature size obtained by our algorithm was slightly higher, there was a significant improvement in classification performance. For DLBCL, CC-DFS selected 1.4% of all features, with the average classification accuracy of 90.37%, which was an 10.34% improvement over PSO-FS. Although the algorithm achieved the same best classification accuracy, our algorithm reduced the number of features by 24.2. For Leukemia1, three-point-one percent of the features were selected, and the average classification accuracy was 94.02%. Compared with PSO-FS, the average classification accuracy was improved by 12.42%, and the best classification accuracy was was improved by 5.28%. For Lung Cancer, the average classification accuracy was 81.40%, which was slightly lower than PSO-FS. but the best classification accuracy increased by 3.98%. Compared with PSO-FS, CC-DFS obtained nine “+” and one “=”, without any “−”.

#### 4.3.4. CC-DFS vs. EPSO

Compared with EPSO, in terms of classification performance, CC-DFS obtained seven “+”, one “−”, and two “=”. The lowest average classification accuracy improvement was 0.65%, appearing on Leukemia1, and the highest improvement was 5.64%, appearing on Leukemia2. The maximum classification accuracy improvement was 5.56%, appearing on Leukemia2. For Brain Tumor1, in terms of average classification accuracy, three-point-seven-eight percent improvement was obtained, and the best classification accuracy increased by 3.91%. Except Leukemia2, on the other datasets, the selected feature subset length was higher than EPSO. In summary, compared with EPSO, at the cost of a small number of features, a good improvement in classification performance was achieved.

#### 4.3.5. CC-DFS vs. PPSO

For eight datasets, CC-DFS achieved better or similar best classification accuracy compared to PPSO. For six datasets, the average classification accuracy of CC-DFS was higher than PPSO. For Lung Cancer, an average of 155.6 features was selected, which was 47.4 fewer than PPSO, while the average classification accuracy was improved by 2.02%, and the best classification accuracy was increased by 5.6%.

#### 4.3.6. CC-DFS vs. CCB-DFS

Compared with CCB-DFS, similar classification performance was achieved on nine datasets. On six datasets, CC-DFS selected fewer features than CCB-DFS. For 9Tumor, in terms of average classification accuracy, one-point-one-two percent improvement was obtained, and the best classification accuracy increased by 3.28%, but the number of selected features dropped by 36.2.

In summary, for 50 comparisons, CC-DFS obtained 31 “+”, 5 “−”, and 14 “=”, which meant that among 45 comparisons, CC-DFS obtained better or similar classification performance, and only five times, the classification performance was lower than the compared algorithm.

### 4.4. Effectiveness of Reset Operation

**Figure 7:**
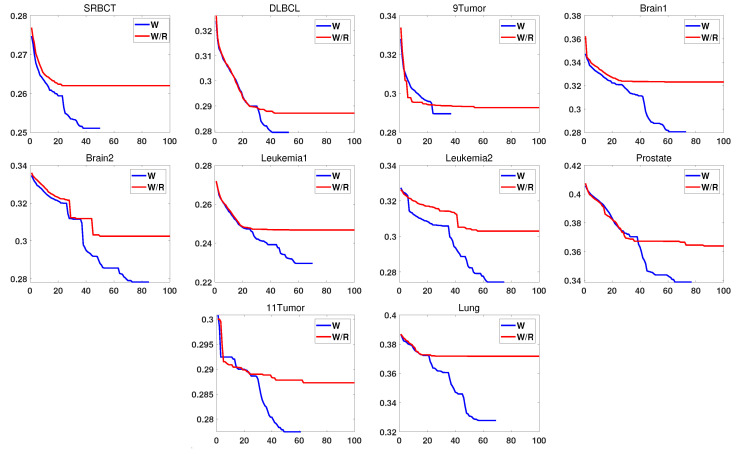
With reset operation and without reset operation.

To verify the performance of the reset operation, in this section, the algorithm with (denoted by W) and without the reset operation (denoted by W/R) were compared on ten datasets. Figure 7 shows the change in the algorithm’s fitness value during the iteration during the update process. Table 4 shows the running time of the algorithm.As shown in Table 4, the running time of the algorithm using the reset operation was much smaller than the running time of the algorithm without the reset algorithm on the 10 datasets. This result was because the reset operation stopped the iteration in advance when the fitness value of gbest was not updated for 11 consecutive times. It can also be seen in Figure 7 that the gbest fitness value of the algorithm using the reset operation was better than the value without the reset algorithm when the iteration was stopped, and because of the early termination mechanism, the number of iterations was also much smaller than W/R. In addition, with fewer iterations, a better fitness value was obtained.

### 4.5. Computational Time

**Figure 8:**
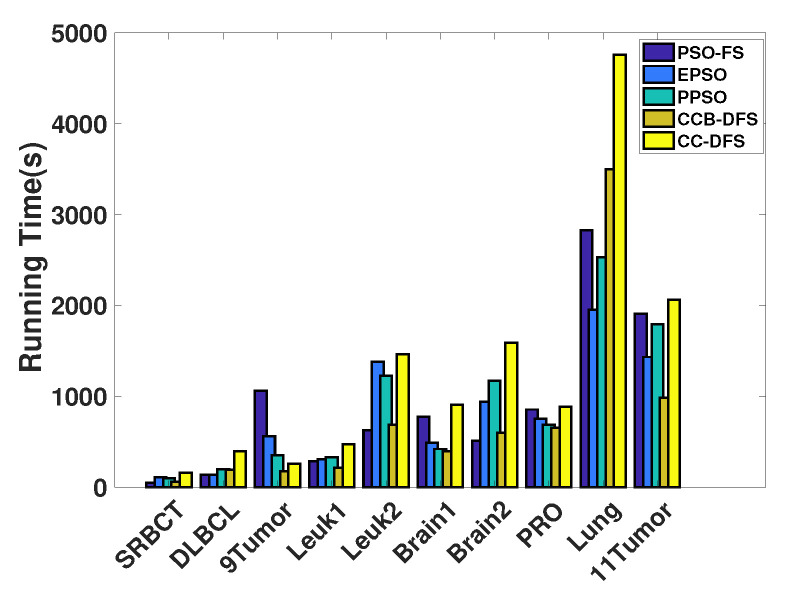
Comparison of the running time.

In this section, we measure the time of feature selection algorithms in Figure 8, since all these approaches were based on evolutionary algorithms and an offline cut-point table.

From Figure 8, we can see that PSO-FS had a shorter running time on most datasets. This was mainly because PSO-FS only discretized the data once before searching while the remaining algorithms needed to discretize the data during the search process. Although the running time of CC-DFS was much higher than that of EPSO and PPSO, its classification accuracy was superior to these two algorithms. As can be seen from Figure 8, the greater the feature size, the greater the gap between the running time of CC-DFS and the running time of EPSO and PPSO. We considered continuous features and selected more features, which in turn led to a much higher running time required by the fitness evaluation process. The reason CC-DFS spent more time than CCB-DFS was that the mutation and crossover operations of the GA consumed more runtime than PSO, but CC-DFS improved the best classification accuracy compared to CCB-DFS. Despite the long running time of our algorithm, considering continuous features significantly improved the classification accuracy.

## 5. Conclusions

In this paper, a new cooperative coevolutionary discretization-based FS method, CC-DFS, was proposed, where both discrete and continuous features were searched to reduce the information loss by only considering the discrete features. During the update process, GA and PSO were applied to search for discrete and continuous feature subsets, respectively. Average classification error and distance measures were used as individual evaluation indicators. The ranking mechanism was introduced to control the mutation and crossover probability of individuals in GA, each dimension of which allowed the mutation and crossover operation with different individuals. Through the distance measure, we selected *N* individuals from discrete features to form a feature subset with continuous features. In addition, a reset operation was used to jump out of the local optimum. Experimental results showed that our method was able to improve the classification accuracy on the benchmark datasets. Compared with using all features, the feature subset selected by CC-DFS could achieve higher classification accuracy. Compared with some state-of-the-art discretization-based methods, it demonstrated the advantages of considering both discrete and continuous features in FS.

## Figures and Tables

**Table 1:** Datasets.

Dataset	# ${}^{1}$ of Features	# of S ${}^{2}$	# of C ${}^{3}$	# of Small	# of Big
SRBCT	2308	83	4	13	35
DLBCL	5469	77	2	25	75
9Tumor	5726	60	9	3	15
Leukemia 1	5327	72	3	13	53
Leukemia 2	11,225	72	3	28	39
Brain Tumor1	5920	90	5	4	67
Brain Tumor2	10,367	50	4	14	30
Prostate	10,509	102	2	49	51
Lung Cancer	12,600	203	5	3	68
11Tumor	12,533	174	11	4	16

${}^{1}$ # means; ${}^{2}$ S means the number of samples; ${}^{3}$ C means the number classes.

**Table 2:** Parameter setting.

Parameter	Setting
Population	No. of features/20 (Limited to 300 and no less than 100)
Maximum iteration	100
c1 and c2	1.49445
β	0.5
Lmin	0.25
Lmax	0.5
Stopping criterion	The fitness value of gbest not improved for 11 iterations

**Table 3:** Experimental results. DMLP, deep multilayer perceptron; FS, feature selection; CCB-DFS, coevolutionary discretization-based using bare-bone PSO FS. (Bold indicates the best value).

Dataset	Method	# of Features	Best (%)	Avg (std)	S
SRBCT	Full	2308.0		87.08	+
	DMLP	128	**100.00**	97.72 (1.67)	+
	PSO-FS	150.0	97.50	91.31 (2.71)	+
	EPSO	137.3	**100.00**	96.89 (1.64)	+
	PPSO	108.5	**100.00**	95.78 (1.96)	+
	CCB-DFS	168.1	**100.00**	**99.26** (0.89)	=
	CC-DFS	220.6	**100.00**	98.90 (1.05)	
DLBCL	Full	5469.0		83.00	+
	DMLP	128	**97.92**	**93.26** (3.35)	−
	PSO-FS	101.8	96.67	80.03 (6.13)	+
	EPSO	42.8	94.17	85.18 (5.46)	+
	PPSO	44.0	94.17	86.22 (3.58)	+
	CCB-DFS	85.6	96.67	90.28 (3.29)	=
	CC-DFS	77.6	96.67	90.37 (3.37)	
9Tumor	Full	5726.0		36.67	+
	DMLP	128	55.97	48.48 (5.61)	+
	PSO-FS	955.0	55.00	45.95 (4.93)	+
	EPSO	138.5	**65.00**	58.22 (3.12)	−
	PPSO	118.1	**65.00**	**59.28** (2.08)	−
	CCB-DFS	314.2	58.20	52.66 (3.64)	=
	CC-DFS	278.0	61.48	53.78 (3.59)	
Leukemia1	Full	5327.0		72.08	+
	DMLP	128	**97.92**	91.64 (3.99)	+
	PSO-FS	150.0	92.22	81.60 (4.72)	+
	EPSO	135.9	95.56	93.37 (1.83)	=
	PPSO	80.4	95.42	**94.37** (1.36)	=
	CCB-DFS	126.8	96.67	94.14 (1.35)	=
	CC-DFS	166.4	97.50	94.02 (1.45)	
Leukemia2	Full	11,225.0		89.44	+
	DMLP	128	96.94	93.48 (1.75)	+
	PSO-FS	150.0	93.89	86.11 (3.97)	+
	EPSO	139.9	94.44	89.93 (2.79)	+
	PPSO	86.7	**100.00**	**96.74** (1.64)	=
	CCB-DFS	346.8	**100.00**	95.17 (2.00)	=
	CC-DFS	131.7	**100.00**	95.57 (2.09)	
Brain Tumor1	Full	5920.0		72.08	+
	DMLP	128	82.57	73.76 (3.69)	+
	PSO-FS	317.3	78.75	71.00 (3.06)	+
	EPSO	150.7	79.17	72.79 (3.48)	+
	PPSO	73.4	82.08	74.40 (3.67)	+
	CCB-DFS	189.5	80.58	75.90 (2.49)	=
	CC-DFS	187.4	**83.08**	**76.57** (3.47)	
Brain Tumor2	Full	10,367.0		62.50	+
	DMLP	128	81.81	73.93 (3.25)	=
	PSO-FS	417.9	82.08	69.11 (5.89)	+
	EPSO	152.8	83.75	70.76 (5.30)	+
	PPSO	66.7	74.58	68.75 (4.24)	+
	CCB-DFS	298.6	**89.17**	**75.75** (4.61)	−
	CC-DFS	138.7	83.75	72.22 (5.01)	
Prostate	Full	10,509.0		85.33	+
	DMLP	128	83.40	74.25 (3.21)	+
	PSO-FS	777.4	90.33	85.20 (2.35)	+
	EPSO	54.9	90.33	83.74 (3.55)	+
	PPSO	65.6	**95.17**	**91.82** (1.77)	−
	CCB-DFS	129.8	92.50	89.06 (2.08)	=
	CC-DFS	180.6	92.17	88.52 (1.63)	
11Tumor	Full	12,533.0		71.42	+
	DMLP	**128**	79.36	73.69 (3.19)	+
	PSO-FS	1638.8	86.07	82.62 (1.70)	+
	EPSO	149.9	83.68	79.29 (2.11)	+
	PPSO	167.0	83.20	76.83 (2.91)	+
	CCB-DFS	1422.4	**89.09**	**85.27** (1.92)	=
	CC-DFS	1890.2	87.77	84.84 (2.47)	
Lung Cancer	Full	12,600.0		78.05	+
	DMLP	128	81.21	73.78 (3.73)	+
	PSO-FS	686.2	85.73	81.72 (2.08)	=
	EPSO	150.8	85.58	80.60 (2.42)	=
	PPSO	203.0	84.11	79.38 (3.26)	+
	CCB-DFS	433.9	86.92	**82.88** (2.13)	=
	CC-DFS	155.6	**89.71**	81.40 (3.10)	

**Table 4:** Running time (s). W, with the reset operation; W/R, without the reset operation.

Dataset	Time (W)	Time (W/R)
SRBCT	157.1	352.2
DLBCL	397.1	782.3
9Tumor	260.4	637.9
Leukemia1	475.1	778.9
Leukemia2	1465.4	2283.8
Brain Tumor1	909.8	1445.1
Brain Tumor2	1591.6	2041.4
Prostate	884.4	1334.8
11Tumor	2062.2	4885.1
Lung Cancer	4756.9	12,075.0
